# Association between anticoagulation strategy and quality of life in chronic hemodialysis patients

**DOI:** 10.1038/s41598-023-42069-2

**Published:** 2023-09-13

**Authors:** Sunny Eloot, Floris Vanommeslaeghe, Iván Josipovic, Matthieu Boone, Wim Van Biesen

**Affiliations:** 1 Nephrology Department, Ghent University Hospital, Corneel Heymanslaan 10, 9000 Ghent, Belgium; 2 Centre for X-ray Tomography, Physics and Astronomy, Ghent University, Ghent, Belgium

**Keywords:** Nephrology, Renal replacement therapy

## Abstract

Prevention of clotting in hemodialysis (HD) is a concern, but tools to monitor anticoagulation strategies as well as data on bleeding and its impact on quality of life (QoL) are scant. In this prospective longitudinal observational study, bleeding tendency in 70 HD patients was scored with ISTH-BAT and HAS-BLED at week 0, 4, and 8. Patient’s limbs were visually scored for bruises and hematomas, and Quality of Life (QoL) was assessed using EQ5D-3L and Visual Analogue Scale (VAS) questionnaires. At week 0, the used hemodialyzer was scanned in a micro-CT scanner to quantify the number of patent fibers. Bleeding scores were 0 [0; 1] and 3 [2; 4] for ISTH-BAT and HAS-BLED, and visual scoring showed 2 [0; 4] bruises/hematomas. QoL was 0.85 [0.77; 1.00] for EQ5D and 70 [60; 80] for VAS. Fiber patency was 81 [70; 90]%, but was not associated with anticoagulation dose (p = 0.103). Patients in the highest tertile of anticoagulation dose had a worse VAS score (p = 0.027), and patients identified as having bleeding tendency by ISTH also had a worse VAS score (p = 0.010). This supports our postulate that in maintenance HD patients the current personal anticoagulation dose regimens may be too high, leading to more mainly minor bleeding that may negatively impact health related quality of life.

## Introduction

Prevention of clotting in the extracorporeal circuit is an important concern in maintenance hemodialysis (HD) patients^1^. Unfractionated heparin and low-molecular-weight heparins (LMWHs) are most popular as anticoagulants in every day clinical practice. As there is no documented objective difference between these approaches in terms of outcome or safety, the preferred choice seems to vary regionally^2^. LMWHs have as advantage the ease and convenience of their administration, but tailoring and monitoring dosing can be cumbersome, increasing the risk of overdosage, drug accumulation and hemorrhage. Management is further hampered by the fact that coagulation disturbances are prevalent in patients on maintenance HD with a subtle balance between a prothrombotic state on the one hand, mostly due to inflammation and underlying endothelial dysfunction, and bleeding risk on the other hand, mostly related to medication and uremic thrombocytopathy^3^.

In addition, until recently, accurate tools to objectively assess coagulation of extracorporeal circuits were lacking. Whereas complete clotting of the extracorporeal circuit is a clear indication of inadequate anticoagulation, more subtle degrees of relevant clotting within the dialyzer are more challenging to identity. Several parameters such as machine transmembrane and venous pressures, and visual inspection of the dialyzer and the extracorporeal circuitry correlate poorly with objective dialyzer fiber clotting^4–7^. Nevertheless, suspected clotting might inspire a nephrologist to increase the dose of anticoagulation in the next dialysis session, and it can be postulated that, over some time, such a practice might lead to overdoses of anticoagulation. In contrast, the side effects of such a practice are much harder to detect clinically, and are thus rarely mediated by a decrease in dose. There is even accumulating data that many patients on maintenance HD receive supra-therapeutic doses of anticoagulation, as even protocols with only a quarter of the regular anticoagulation dose seem not to result in any clotted dialyzer or circuit^5,8^.

An objective online tool to quantify coagulation during maintenance hemodialysis in order to better optimize or individualize the anticoagulation dose is however lacking. Recently, exact quantification of dialyzer clotting using micro-CT scanning of dialyzers post dialysis has been introduced as the gold standard technique, but this tool is not yet available for clinical practice^7,9^.

Quality of life and life participation are important for patients on maintenance hemodialysis^10^. Adverse bleeding events however were found associated with a lower Health Related Quality of Life (HRQoL) in patients on antithrombotic agents^11^. Since patients on maintenance HD have an increased risk to fall, they are at risk for serious bleeding complications in case of over-aggressive anticoagulation. The fear for falling might further be enhanced by a perceived higher bleeding tendency, resulting in a decreased activity and mobility, in itself contributing to decreased life participation and decreased QoL. Furthermore, data on major bleeding complications and their impact on HRQoL in maintenance HD patients are largely unknown. In a meta-analysis exploring additional risk of anti-platelet therapy in hemodialysis, there were 102 major bleeding events in 733 patients (14%) in the control group, which was not different in the intervention group^12^. For minor bleeding events, data are even more absent.

We postulate that a substantial part of patients on maintenance HD are over-anticoagulated, and therefore have a high incidence of bleeding events, with a negative impact on their HRQoL.

The present observational study therefore aimed at mapping bleeding incidence (by visual scoring and questionnaires), quality of life (by questionnaires), and dialyzer fiber blocking (as objectively obtained by µCT scanning), and relate these to anticoagulation doses in a population of in-center maintenance HD patients.

## Results

**Figure 1:**
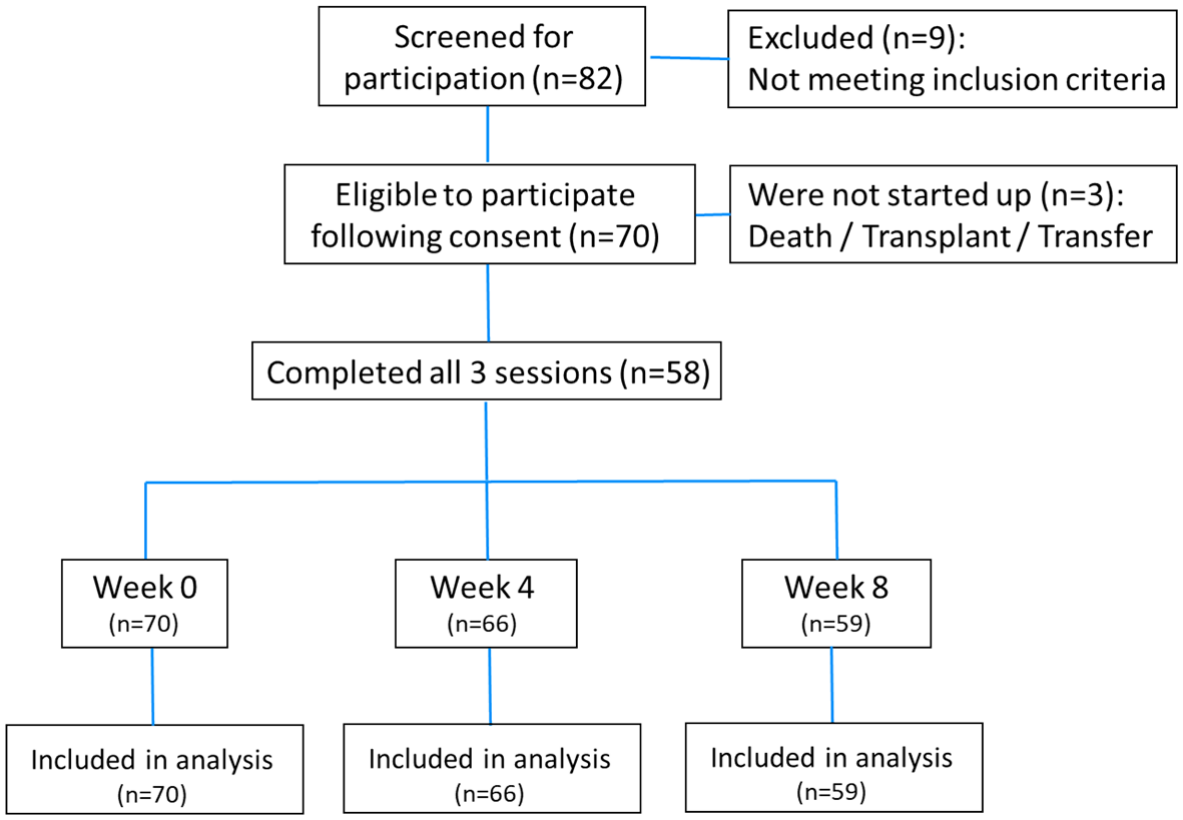
Patient inclusion flowchart.

From the 73 eligible patients, 70 completed all tests in week 0 and 58 of them completed all tests in week 0, 4, and 8 (flowchart in Fig. 1). Relevant demographic, clinical, and dialysis data of the patient population at baseline are summarized in Table 1. The 70 included patients (age 60.0 ± 17.9; 38 male) had stable single needle (n = 17) or double needle (n = 53) dialysis through an arterio-venous fistula (n = 49) or a well-functioning tunneled central venous catheter, i.e. Hemostar 14.5F (Bard, Salt Lake City, UT) (n = 10), Palindrome 14.5F (Medtronic, Minneapolis, MN) (n = 8), or Tesio (Medcomp, Harleysville, PA) (n = 3). Patients were treated with regular hemodialysis (n = 52) or post dilution hemodiafiltration (n = 18), all with high flux dialyzers, i.e. FX800 Cordiax (Fresenius Medical Care, Bad Homburg, Germany) (n = 43), Theranova 400 (Baxter, Deerfield, IL) (n = 18), ATA™ Solacea™ 19H (Nipro, Osaka, Japan) (n = 8), and Evodial 1.3 (Baxter, Deerfield, IL) (n = 1).

**Table 1:** Demographic and clinical data of the patient population at baseline.

Gender (M/F)	38 M/32 F
Age (years)	60.0 ± 17.9
Dry body weight (kg)	76.4 ± 18.9
Renal disease	Glomerulonephritis (n = 18); diabetes mellitus (n = 11); renal vascular disease (n = 10); ADPKD (n = 9); other (n = 22)
Vascular access	Arterio-venous fistula (n = 49); central venous catheter (n = 21)
Anticoagulation dose	Tinzaparin 3500 (n = 17)
	Enoxaparin 20 mg (n = 12); 40 mg (n = 28); 60 mg (n = 7); 80 mg (n = 1)
	Fondaparinux (n = 1)
	No anticoagulation (n = 4)
Platelet inhibitors	Acetylsalicylic acid (n = 26); clopidogrel (n = 6); dual antiplatelet (n = 4); coumarin (n = 2)
Hb (g/dL)	10.9 ± 1.4
Platelets count (10^3^/µL)	217 ± 75
aPTT (s)	34.5 [32.6; 38.1]
INR (–)	0.98 [0.93; 1.03]
CRP (mg/L)	4.2 [1.4; 10.9]

*Hb* hemoglobin, *aPTT* activated partial thromboplastin time, *INR* international normalized ratio, *CRP* C-reactive protein, *ADPKD* autosomal dominant polycystic kidney disease.

Anticoagulation of the extracorporeal system was achieved with Low Molecular Weight Heparin (LMWH) (i.e. enoxaparin n = 48 and tinzaparin n = 17), direct anti-Xa inhibitors (fondaparinux, n = 1) or no systemic anticoagulation (n = 4) (Table 1). In the 4 months prior to the study, six patients (8.6%) had an increase in anticoagulation dose, and only one patient had a reduction in dose. Of the 70 included patients, 36 were on platelet inhibitors (acetylsalicylic acid only n = 26; clopidogrel only n = 6; and dual antiplatelet therapy n = 4) and 2 on oral anticoagulation, of which one also on antiplatelet therapy.

**Figure 2:**
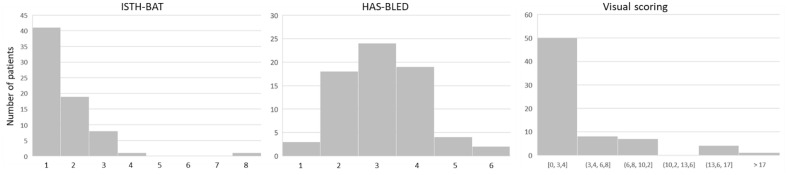
Histogram of ISTH-BAT, HAS-BLED, and visual scoring of hematomas in week 0.

Table 2 shows the assessment of bleeding and Quality of Life scores for the three test sessions. There was no variation over time for the ISTH-BAT, HAS-BLED, EQ5D, and VAS score (all p = NS). At week 0, bleeding scores were 0 [0; 1] for the ISTH-BAT and 3 [2; 4] for the HAS-BLED questionnaires, and the number of bruises and hematomas as counted during the visual scoring was 2 [0; 4] (Fig. 2). Quality of life was scored 0.852 [0.770; 1.000] for the EQ5D and 70 [60; 80] for the VAS questionnaire.

**Table 2:** Bleeding and quality of life scores.

	Bleeding scores			QoL scores	
	ISTH-BAT	HAS-BLED	Visual scoring	EQ-5D score	VAS score
Week 0	0 [0; 1]	3 [2; 4]	2 [0; 4]	0.852 [0.770; 1.000]	70 [60; 80]
Week 4	0 [0; 1]	3 [2; 4]	2 [1; 6]	0.843 [0.775; 1.000]	70 [62; 80]
Week 8	0 [0; 1]	3 [2; 4]	1 [0; 2]	0.897 [0.775; 1.000]	70 [60; 80]

Median [25 pct; 75 pct].

**Figure 3:**
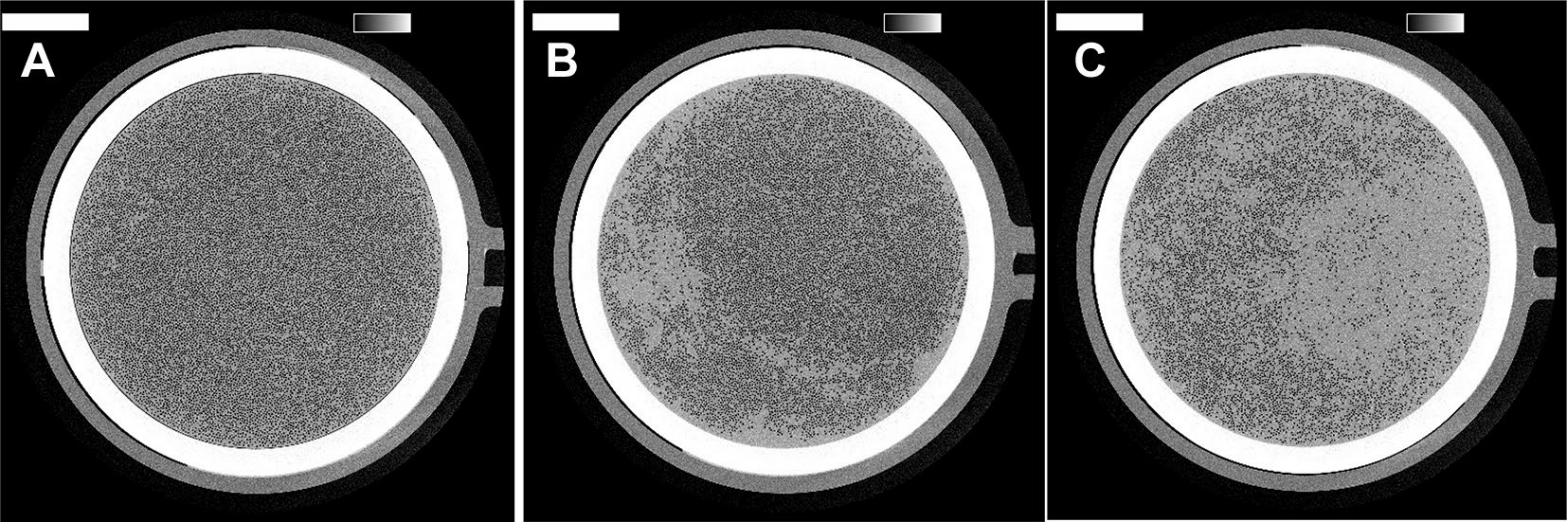
Representative cross-sections of dialyzer outlet potting of FX800 Cordiax dialyzer, illustrating nearly no fiber clotting (~ 99% fiber patency—(**A**)), average fiber clotting (~ 79% fiber patency—(**B**)), and severe fiber clotting (~ 47% fiber patency—(**C**)). The greyscale range is from 0 to 0.5 cm^−1^ and the scale bar denotes 10 mm.

Representative cross-sections of dialyzer outlet potting are presented in Fig. 3, illustrating nearly no fiber clotting (~ 1% of fibers), average fiber clotting (~ 21% of fibers) and severe fiber clotting (~ 53% of fibers), respectively. The percentage of open fibers in the dialyzers, of which minimum 90% of the lumen of the fibers is open, is 81 [70; 90]%. Thus, even with this very strict criterium to declare a fiber ‘open’, still 3/4th of the patients achieved this high target for more than 70% of the dialyzer fibers.

**Figure 4:**
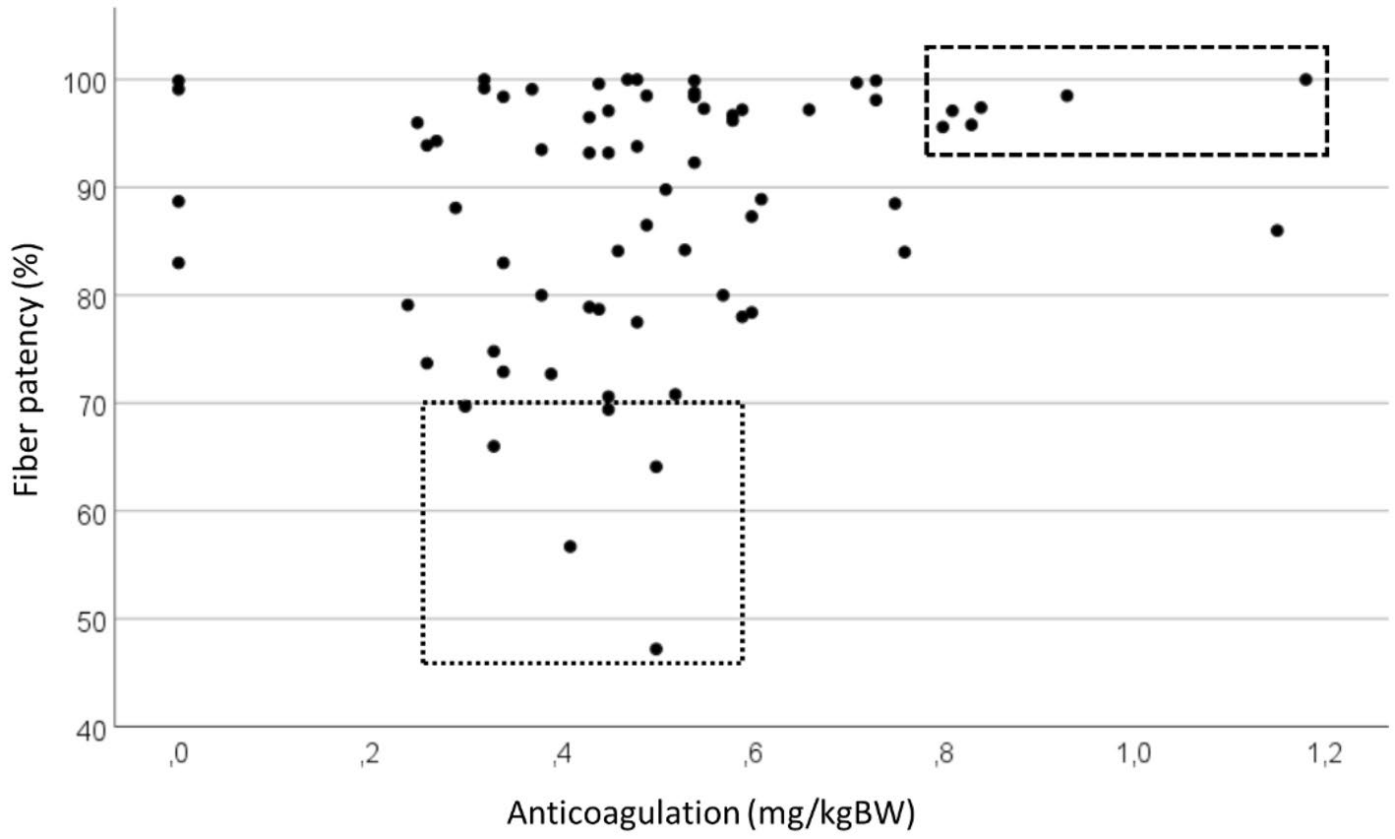
Relation between the dialysis anticoagulation dose and the percentage of patent fibers post dialysis.

No correlation was found between the dose of dialysis anticoagulation normalized for body weight and the percentage of open fibers post dialysis (P = 0.103), mainly because so many patients did not show any degree of fiber clotting (Fig. 4). Also, conversion between different LMWHs is generally based on anti-Xa activity, accepted being the primary biomarker for anticoagulant effects of LMWHs and to determine their strength^13^. Additionally to their anti-Xa action, LMWHs can however to a variable degree inhibit factor IIa and induce tissue pathway factor inhibitor release from endothelial cells. This variability and differences in pharmacokinetic properties might influence the effect of different LMWHs, and thus the studied correlation. Fiber patency was also not different depending on the number of platelet inhibitors and/or oral anticoagulation (P = 0.597).

At week 0 and among the different QoL and bleeding parameters, correlations were observed between EQ5D and VAS scores (P < 0.001), and a trend was seen between ISTH score and visual scoring (P = 0.054) and between HAS-BLED and visual scoring (P = 0.077). But, cross-sectionally at week 0, neither the bleeding scores of ISTH, HAS-BLED and visual inspection, nor the QoL as assessed by EQ5D and VAS were different according to the percentage patent fibers and the dialysis anticoagulation dose.

Accounting for the longitudinal data, patients in the highest tertile of anticoagulation dose adjusted for body weight had a worse VAS score (i.e. 63) as compared to those in the lowest tertile (i.e. 73) (p = 0.027, mean difference 9.8 points, 95%CI [1.1; 18.4]). And patients identified as having versus not having a bleeding tendency by the ISTH had a worse VAS score (i.e. 67 versus 72), even after adjusting for dialysis anticoagulation dose (p = 0.010, mean difference 5 points, 95%CI [1.2; 8.9]). Both other bleeding scores (i.e. HAS-BLED and visual inspection) showed no association with VAS, and none of the three bleeding scores showed differences according to the EQ5D score.

**Figure 5:**
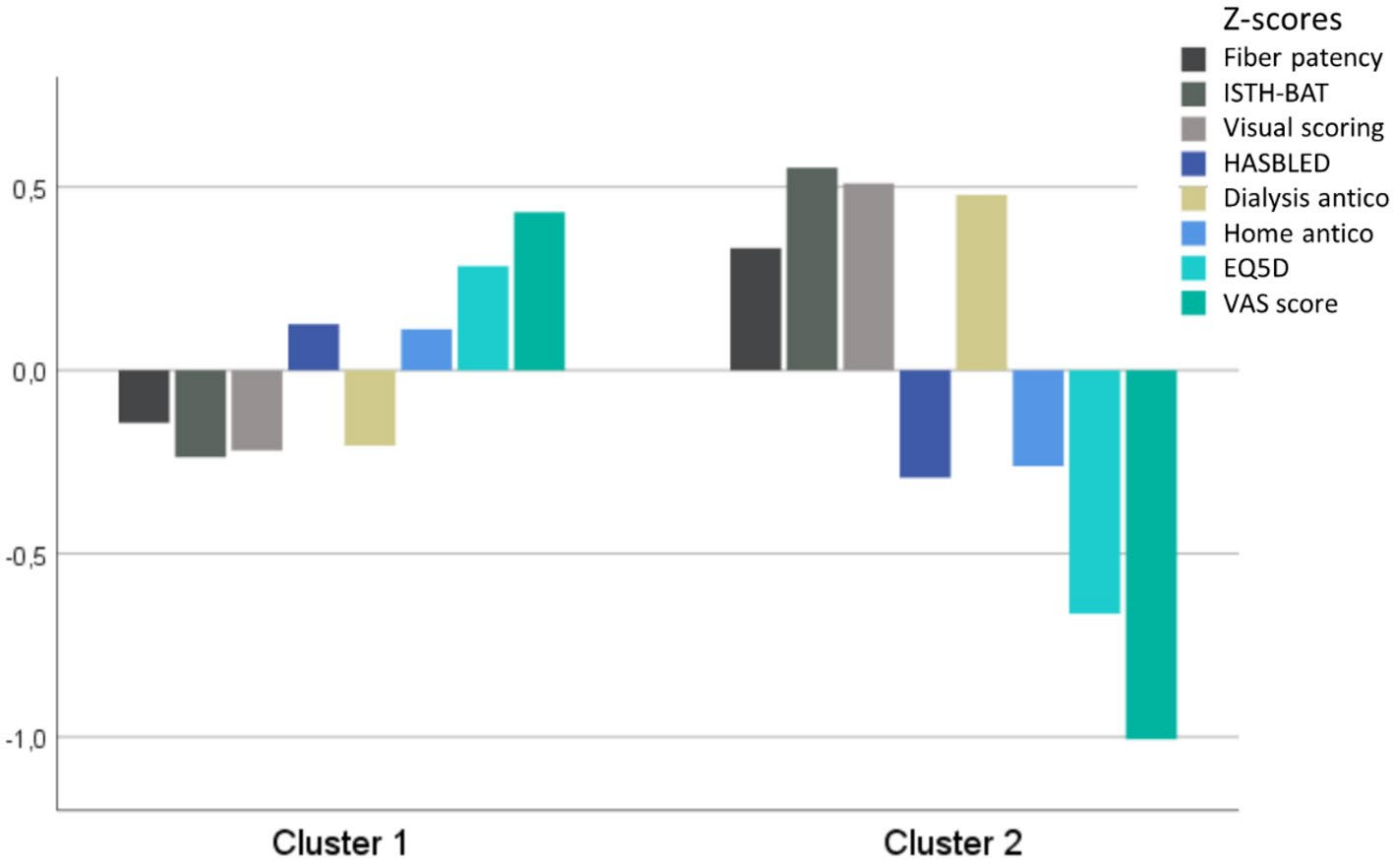
Cluster analysis.

Cluster analysis divides the patients in two groups (Fig. 5) with 49 patients in cluster 1, characterized by lower dialysis anticoagulation dosing (P = 0.008), lower fiber patency (borderline P = 0.067), lower bleeding scores for ISTH (P = 0.002) and visual scoring (P = 0.004), and higher Quality of Life as scored with EQ5D (P < 0.001) and VAS (P < 0.001), as compared to the 21 patients of cluster 2.

No differences in fiber patency were observed between the two types of vascular access, i.e. arterio-venous fistula versus central venous catheter, neither among the types of anticoagulation, i.e. tinzaparin, enoxaparin, fondaparinux, and no anticoagulation. Anticoagulation dosing at the dialysis start was however higher in female patients, even when normalized for body weight (P < 0.001).

## Discussion

In the present study, none of the sessions was interrupted because of clotting and the degree of dialyzer fiber blocking was on average very limited when objectively quantified, whereas a substantial degree of minor bleeding complications was registered. This supports our postulate that in maintenance HD patients the current personal anticoagulation dose regimens may be too high, leading to mainly minor bleeding that may negatively impacthealth related quality of life. On the other hand, a small number of patients has substantial fiber clotting that apparently was not captured by the treating team. All these suggest there is an urgent need for an objective tool to evaluate clotting during the dialysis session so that anticoagulation can be individually tailored.

Effective anticoagulation of the extracorporeal system is essential for achieving adequate dialysis sessions. Unfortunately, good tools to evaluate degree of anticoagulation during a dialysis session are lacking, and currently used measures such as venous pressure or transmembrane pressure are unreliable^7^. Similarly, post dialysis, it is rather cumbersome, as with the µCT dialyzer scanning technique, to assess whether or not appropriate anticoagulation was achieved during the session.

The doses of anticoagulation are mostly determined based on rules of thumb and gut feeling, and few units have or apply standardized evidence based operating procedures to tailor the dose of anticoagulation. For example, in our study, we found that anticoagulation dose was higher in females, most likely because, in our center, weight is not accounted for when deciding on dose and because the commercially available doses cannot easily be customized to fit each individual. Furthermore, in the 4 months preceding the study, dose of anticoagulation was only rarely adapted, and if it was adapted, it was six times more likely to be increased than to be downscaled. Most likely, this is due to the fact that consequences of enhanced clotting are directly experienced during or after the dialysis session, whereas consequences of overanticoagulation are mostly occurring in the interdialytic interval, and are not picked up by the treating team. The majority of the scanned dialyzers shows nearly no clotted fibers, even when very strict limits are applied to consider a fiber as open. Some of these patients do however receive high doses of anticoagulants (i.e. patients within the dashed rectangle in Fig. 4), indicating that dose could probably be reduced without problems.

On the other hand, some dialyzers showed substantial fiber clotting, and in these patients (i.e. patients within the dotted square in Fig. 4), it might be indicated to increase anticoagulation dose. Some of these patients receive average or rather low doses of anticoagulation and might thus benefit from receiving a higher dose. All these suggest that current approaches to anticoagulation are suboptimal, and that a regular assessment of fiber clotting using an objective tool is probably necessary to optimize individual patient tailored anticoagulation dose.

Although major bleeding was rather rare, minor bleeding complications were frequent to almost omnipresent when objectively asked and searched for. There was a clear relationship between QoL as assessed by VAS and the ISTH questionnaire about bleeding incidence. Furthermore, a cluster analysis (see Fig. 5) identified a group of patients in whom higher than average weight adjusted doses of anticoagulation were coupled with more bleeding complications and lower quality of life, despite higher objective measures of fiber patency.

Life participation is an important outcome for patients, and is directly coupled to QoL^14^. It can be speculated that the negative consequences of overanticoagulation in terms of hematomas and bleeding, have a negative impact on life participation, as they impede patients to do activities and participate in social life. Patients on dialysis have an enhanced risk of falls^15^, and it has been demonstrated earlier that it is especially the fear for falling that has a negative impact on quality of life in these patients, as this impedes patients from doing activities that used to give meaning to their life. A comparable mechanism might be at play with overanticoagulation. However, it could be argued that more frail patients, with higher likelihood of inflammation such as in the malnutrition-inflammation-atherosclerosis (MIA) syndrome need higher doses of anticoagulation, but have a lower quality of life due to their MIA syndrome. However, based on the cluster analysis, this explanation, though plausible, is not compatible with the observation that these patients have better than average fiber patency, and making it more likely that they in fact unnecessarily receive too high doses of anticoagulation.

In conclusion, there is a need for tools to individually tailor anticoagulation for maintenance hemodialysis patients. Such tools would help to avoid overanticoagulation, and to potentially limit bleeding incidence and lowering of health related quality of life.

## Methods

### Patients

This single center prospective longitudinal observational study included all stable patients on regular maintenance hemodialysis (HD). The protocol adhered to the Declaration of Helsinki, was approved by the institutional research committee (Ethical Committee—Ghent University Hospital, BC 11802—B6702022000121—19/04/2022), and was registered in https://www.clinicaltrials.gov (NCT05365542—19/01/2022—‘Anticoagulation in Chronic Hemodialysis Patients Versus Hemodialyzer Fiber Patency, Bleedings and Quality of Life’). Written informed consent was obtained from all included patients.

### Study setup

The study was run over an 8 week period, with three monitoring moments for each patient (i.e. week 0, 4, and 8). During the entire study period, patients were dialyzed with their regular dialyzer and dialysis machine and with their typical dialysis settings and anticoagulation dose.

On week 0, 4 and 8, quality of life status and bleeding tendency were assessed using questionnaires (see below), and limbs of patients were visually scored for bruises and hematomas. After the first midweek dialysis session (week 0), the used hemodialyzer was rinsed with the standard rinsing procedure (i.e. 300 mL), dried in both blood and dialysate compartment with positive pressure ventilation during 12–24 h, and stored at 5 °C until scanning in a micro-CT scanner in order to quantify the number of patent fibers^7^.

### Data collection

From the patient's medical record, the following data were prospectively retrieved: demographic data (i.e. age, gender, body weight, dialysis vintage, vascular access), blood results related to clotting [i.e. hemoglobin, number of platelets, activated Partial Thromboplastin Time (aPTT), International Normalized Ratio (INR), C-reactive protein (CRP)], and relevant medication with a potential impact on coagulation (i.e. antithrombotic drugs, platelet inhibitors). For each patient, history of anticoagulation prescribed for dialysis was retrieved and recorded.

More granular data of the midweek dialysis session of week 0 were collected, i.e. dialysis machine type, hemodialyzer type, dialysis mode, flows (blood, dialysate and substitution), ultrafiltration rate, type and dose of anticoagulation.

### Questionnaires and visual scoring

To score the tendency for bleeding the International Society of Thrombosis and Haemostasis Bleeding Assessment Tool (ISTH-BAT) was questioned about the past 4 weeks^16^, and the Hypertension, Abnormal Renal/Liver Function, Stroke, Bleeding History or Predisposition, Labile INR, Elderly, Drugs/Alcohol Concomitantly (HAS-BLED) score was derived from patient’s medical record^17^. As the ISTH-BAT was developed to identify patients with intrinsic bleeding disorders, including thrombocytopathy, we used it to identify patients at higher risk of bleeding^16^. Underlying idea was that especially thrombocytopathy is prevalent in patients on maintenance HD, due to several reasons. The HAS-BLED score was used to assess overall bleeding risk^17^.

Health related quality of life (HRQoL) was assessed using the EuroQoL 5D (EQ-5D-3L) questionnaire. This is a validated generic questionnaire consisting of five dimensions (i.e. mobility, self-care, daily activities, pain/discomfort, and anxiety/depression) with each three severity levels (i.e. no problems, some problems and extreme problems) to assess HRQoL. The resulting health state or EQ-5D utility index ranges from 0 (worst possible health) to 1 (best possible health) and was calculated by applying the appropriate value set as published by the EuroQoL group^18^. The patient was also asked to score his/her health on the EQ-5D Visual Analogue Scale (EQ-5D VAS) ranging from 0 to 100, corresponding to the worst *versus* best imaginable health state^19^.

Finally, on each monitoring session, a dedicated nurse performed a visual inspection of the patient’s limbs and quantified the number of bruises and hematomas.

### Micro-CT scanning and coagulation quantification

Micro CT scanning was performed at week 0, as described earlier. In short, the used and dried dialyzer is vertically mounted on a rotating disc in front of the X-ray bundle. Scans of the dialyzer outlet potting are made every 0.15° with 2401 projections during 500 ms each. Octopus Reconstruction software package as licensed by XRE, a Ghent University spin-off company, was used to reconstruct the raw projection data into 2D visualizations of cross-sections of the hemodialyzer. Non-blocked fibers were counted in the central cross-section of the dialyzer outlet potting, using an open-source platform for biological-image analysis (ImageJ 1.51 H, NIH, Bethesda, USA). A fiber was assumed ‘open’ when 90% of the cross-section of a non-used fiber was open. Comparing the number of open fibers in the tested dialyzer with the total number of fibers as previously measured in three non-used dialyzer samples, provides an objective estimate of the percentage of fiber patency^7^.

### Data and statistical analysis

Statistical analyses were performed using SPSS (version 27, SPSS Inc, Chicago, USA). Continuous variables were summarized as mean ± SD or median (25th percentile; 75th pct). Normality was checked by Shapiro–Wilk. Correlations between continuous variables were performed using Spearman correlation. Differences between groups (tertiles or categories) are checked with Kruskal Wallis for continuous variables and Chi square test for categorical variables. Categorical variables were herewith discretized in groups: i.e. ISTH category 0 (for ISTH score = 0) or 1 (ISTH score > 0); HAS-BLED category 0 (score 0), 1 (score 1–2), 2 (score 3–4), or 3 (score > 4); and visual scoring category 0 (no bruises), 1 (number of bruises < median), 2 (number of bruises: median to 75th percentile), or 3 (number of bruises > 75th percentile). Linear mixed models with random intercept for patient were performed using the longitudinal data, and k-Means cluster analysis was performed with all measured parameters.

## Data Availability

All data generated or analyzed during this study are included in this published article.
